# Tolerability and Safety of Sublingual Immunotherapy in Patients with Tree Pollen Allergy in Daily Practice—An Open, Prospective, Non-Interventional Study

**DOI:** 10.3390/jcm12175517

**Published:** 2023-08-25

**Authors:** Christoph Owenier, Cornelia Barnowski, Margret Leineweber, Donghui Yu, Marjan Verhagen, Andreas Distler

**Affiliations:** 1 HAL Allergie GmbH, 40213 Düsseldorf, Germanyadistler@hal-allergy.com (A.D.); 2 HAL Allergy BV, 2333 CH Leiden, The Netherlands; dyu@hal-allergy.com (D.Y.); mverhagen@hal-allergy.com (M.V.)

**Keywords:** sublingual immunotherapy (SLIT), birch/tree pollen, non-interventional study, safety, tolerability, patient satisfaction with SLIT

## Abstract

To investigate the tolerability and safety of two sublingual tree pollen extracts approved in 2018, a non-interventional study (NIS) was performed. This NIS was an 8-month observational study conducted at 84 sites throughout Germany. Study participants received either a sublingual liquid allergen extract of birch pollen (SBPE) or a liquid allergen extract consisting of a mixture of birch, hazel, and alder tree pollen (STPE). Data from 432 patients were analyzed for the occurrence of adverse events and patient compliance. At least one local reaction occurred in 69 (22.2%) patients, whereas systemic reactions were only observed in 27 (6.3%) patients. STPE-treated patients developed systemic reactions more frequently than SBPE-treated patients (SBPE: 9 (4.3%) vs. STPE: 18 (8.0%)). Only one patient developed a systemic grade III reaction. Severe systemic grade IV reactions were not observed. A total of 348 (98.6%) of the patients who completed all visits were satisfied or very satisfied with the sublingual immunotherapy (SLIT), and 322 (71%) patients completed all visits. Both investigated products were well tolerated by the patients and demonstrated a good safety profile. AEs were observed less frequently than in the preceding clinical phase III trial, and no new safety concerns were identified.

## 1. Introduction

Allergen immunotherapy (AIT) is an effective way to treat allergic rhinitis and rhinoconjunctivitis with or without allergic bronchial asthma in patients sensitive to aeroallergens [1,2]. An important source of airborne spring allergens in Germany is tree pollen (hazel, alder, birch). Patients with seasonal allergies often have a positive skin prick test (SPT) or elevated specific IgE levels against different types of tree pollen [3]. 

The Therapy Allergens Ordinance (“Therapieallergene-Verordnung [TAV]”), which has been in force since 14 November 2008 [4], requires that immunotherapy products containing the tree pollen allergens from birch, alder, and hazel have marketing authorization in Germany. Within the TAV study program, a phase II dose-range-finding (DRF) study and a one-year pivotal phase III study are required to apply for marketing authorization. The DRF study with sublingual birch pollen extract (SBPE) was performed on 269 adults suffering from birch pollen-induced allergic rhinitis. The patients were randomized to placebo or SBPE 3333, 10,000, 20,000, or 40,000 allergy units native (AUN)/mL. Compared with the other dosage groups, the largest reduction of allergy-related symptoms was observed in the 40,000 AUN/mL group [5]. In the subsequent pivotal phase III study, the efficacy and safety of treatment with SBPE 40,000 AUN/mL were compared with placebo. The difference in the combined symptom medication score (CSMS [6]) showed a statistically significant (*p* < 0.0001) and clinically relevant (32%) reduction in the CSMS in the treatment group of SBPE compared with placebo after 3–6 months of pre-seasonal and 3 months co-seasonal treatment [7]. The total treatment period was between 6 and 9 months. The pivotal study with SBPE 40,000 AUN/mL demonstrating clinical efficacy was finalized successfully in 2016, and marketing authorization for patients ≥ 18 years was granted by the Paul-Ehrlich-Institute (PEI) in August 2018. At the same time, the sublingual tree pollen extract (STPE) 40,000 AUN/mL, containing a mixture of birch, hazel, and alder pollen, was granted marketing authorization for patients ≥ 18 years old because the allergens of the three tree pollen species are highly homologous.

The non-interventional study (NIS) described here aimed to evaluate the safety and tolerability of SBPE and STPE preparations by measuring the frequency and intensity of local and systemic reactions in a large patient population during an 8-month treatment period in daily practice.

## 2. Materials and Methods

### 2.1. Study Design

The study was performed as an open-label, prospective, multicenter, post-authorization safety study/NIS according to §63f paragraph 1 of the German Drug Law and was approved and conducted in Germany. A total of 84 centers were involved in this NIS, which recruited 437 patients. Patients were studied over a treatment period of 8 months.

### 2.2. Ethical Aspects

The NIS was registered with the German regulatory authority (Paul-Ehrlich-Institute, Langen, Germany). Furthermore, the study was carried out in accordance with international and applicable German regulations. Written informed consent was obtained from each patient prior to participation in the study. To protect the privacy of participants, the data collection was carried out in accordance with the European data protection law (EU2016/679) (Datenschutz-Grundverordnung (DS-GVO)).

### 2.3. Procedures

Patients were recruited between autumn 2018 and December 2019 by allergologists. The NIS was conducted at 84 centers across Germany, which recruited a total of 437 patients. Patients were enrolled in the study after the decision for treatment with birch or tree pollen sublingual immunotherapy (SLIT) had already been made. The patients participated for 8 months in the study. This observation period started with a screening visit followed by initiation of the treatment with SBPE or STPE two to three weeks later at the physician’s office. Patients eligible for this study were female and male (≥18 years) with moderate/severe seasonal allergic rhinitis/rhinoconjunctivitis, with or without concomitant mild to moderate controlled allergic asthma. Tree-pollen-induced rhinitis/rhinoconjunctivitis was confirmed by a positive SPT and/or positive serum test for IgE specific for tree pollen (hazel, alder, birch) (>0.7 U/mL), positive serum test for Bet v 1 specific IgE, and a positive nasal or a conjunctival provocation test for tree pollen (hazel, alder, birch). Exclusion criteria were as follows: contraindications according to the summary of product characteristics (SPC) [8] of SBPE/STPE, uncontrolled asthma (forced expiratory volume in 1 s (FEV1) <70% of predicted value), or asthma exacerbation within the last 3 months; pregnancy; participation in a clinical trial with SLIT or subcutaneous immunotherapy (SCIT) during the duration of the study; lack of cooperation or compliance according to the investigator’s opinion. 

Overall, 5 visits at the physician’s office were planned: a screening visit (V1), an administration visit (V2), two maintenance visits (V3, V4), and an end-of-study visit (V5). The investigators completed an electronic case report form (eCRF) for each patient during every visit. At the first visit, V1, the investigators checked the in- and exclusion criteria and documented demographic data, medical history, concomitant diseases, and/or medication usage. Moreover, the informed consent was signed and SBPE or STPE was prescribed at V1. For new patients, a diagnostic test (SPT and/or serum IgE) for the most common allergens, as well as hazel, alder, and/or birch pollen, was performed to confirm a tree pollen allergy. Diagnostics were not performed if tree pollen allergy had been diagnosed within the last year prior to the initiation of sublingual therapy. During the second visit, V2, the SLIT with SBPE or STPE was initiated and the date and time of the first use as well as early local or systemic reactions were documented. Since most of the adverse events (AEs) occur early after initiation of the SLIT, the patients were additionally provided with diaries at the second visit to document all AEs during the first 3 weeks and the individual maintenance dose that had been achieved. During the following two maintenance visits, V3 and V4, treatment compliance and occurrence of AEs were checked and documented by the investigators. Furthermore, a new vial of SBPE or STPE was prescribed. During the last visit, V5, patients were asked about their treatment satisfaction, and AE data were collected (Table 1). 

According to the SPC of SBPE and STPE, it is recommended to start the sublingual therapy outside of the pollen season and to continue treatment during pollen flight. However, it is also possible that treatment can be started within the pollen season if the patient is closely monitored, as temporary dose reduction is possible in case of side effects [8]. In this NIS, the majority of patients (309 patients) started therapy outside of the tree pollen season (June–December). Only a subgroup of 123 patients started the treatment with SBPE or STPE during the tree pollen season (January–May). We observed a comparable incidence of adverse events in both groups since 78 (25.2%) patients who started therapy outside of the pollen season and 24 (19.5%) patients who started treatment during the tree pollen season experienced at least one AE.

### 2.4. Study Treatment

Patients were treated with SBPE (SUBLIVAC^®^ Birch (HAL Allergy BV, Leiden, The Netherlands)) or STPE ((SUBLIVAC^®^ Trees (HAL Allergy BV, Leiden, The Netherlands)) containing 40,000 AUN/mL available as registered AIT products on the German market. Both allergen products are liquid preparations for sublingual administration containing allergens extracted from birch pollen or birch, hazel, and alder pollen, respectively. SBPE and STPE are indicated for the treatment of immediate types of allergic disorders (IgE-mediated), such as allergic rhinitis, allergic conjunctivitis, and controlled allergic bronchial asthma, triggered by exposure to tree pollen (birch, hazel, and alder). The SLIT medication was ordered according to the regular on-site procedures, issued to the patient, and stored at the patient’s home. Patients received SLIT according to the SPC [8] with initial administration under the guidance of the physician on-site. Patients started the treatment with one drop and added one drop each consecutive day until the maintenance dose of five drops per day was reached. Five drops contain approx. 100 µg Bet v 1 [7]. Drops were held underneath the tongue for 2–3 min before swallowing. In case of local or systemic reactions, dose adjustments according to the SPC [8] were performed. If local or systemic reactions in response to allergen administration occurred or rescue treatment was necessary, the physician kept a record of such treatments. Recommendations for treatment adjustments were given in the SPC [8]. If symptomatic relief was required for allergic rhinitis/rhinoconjunctivitis complaints, patients used symptomatic medication according to the ARIA guidelines [2]. The treating physician decided which treatment the patient received and documented it in the eCRF.

### 2.5. Safety and Tolerability Assessment

Local reactions were graded according to the World Allergy Organization Taskforce report [9] into mild, moderate, and severe cases by the investigator. Systemic reactions were graded according to the World Allergy Organization Taskforce report grading system [10] into grade I–V reactions. All reported AEs were documented by the physicians using an eCRF that was provided via an internet portal.

### 2.6. Statistical Analysis

Data are presented as total numbers and as percentages. For the description of the study population, mean values are given. Patients with known administration of at least one drop of the investigational medicinal products were included in the analyses (All-Patients-Treated approach). Continuous data were summarized with descriptive statistics (number, mean, median, interquartile range). Categorical data were presented using frequency counts and percentages.

## 3. Results

### 3.1. General Epidemiological Data

This NIS was conducted at 84 centers across Germany. Of the 437 patients enrolled during the NIS, 2 were younger than 18 years, 2 had no signed informed consent, and 1 patient had been given an unknown treatment which led to exclusion from the study leading to a final number of 432 evaluable patients (Table 2). The average age of the patients was 44.3 ± 14.6 years. The gender distribution was comparable and the number of patients receiving STPE and those receiving SBPE was balanced (STPE: 51.9% vs. SBPE: 48.1%). A total of 11.8% of the patients had a known history of asthma.

Different numbers of patients are shown in the following analyses since only patients for whom data were available were included.

### 3.2. Number of Patients Who Reached the Maintenance Dose of Five Drops

All study participants took the first drop of the medicinal product at the physician’s office under the supervision of the physician. As shown in Table 3, 96% of the patients reached the maintenance dose of 5 drops. Only 4% of the patients were not able to reach the maintenance dose during the 8-month treatment period. A total of 94.3% of the patients reached the maintenance dose within 5 days.

### 3.3. Study Adherence and Treatment Compliance

**Figure 1:**
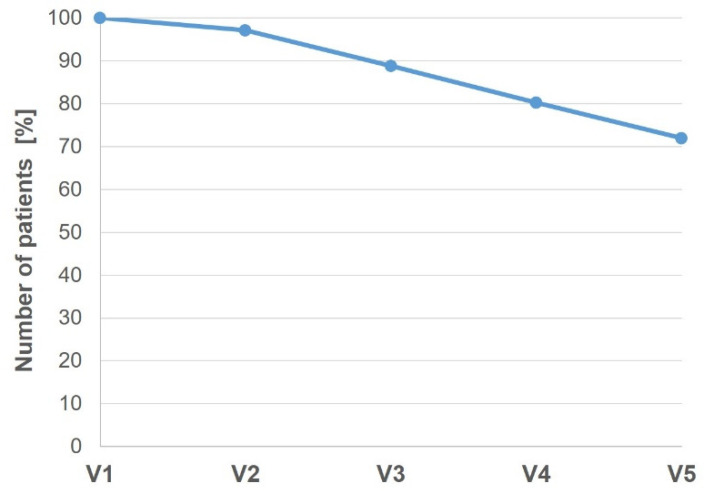
Treatment compliance during the 8-month treatment period at visits V1–V5.

An important factor for the successful outcome of SLIT treatment is the compliance of patients [11,12]. The treatment compliance during SBPE or STPE treatment in this NIS is shown in Figure 1. Out of 432 patients, 320 (72%) patients completed all visits. Before treatment with SBPE or STPE was started, 13 patients (3%) dropped out after V1. For the following visits, a dropout rate of around 8% was observed, since 37 (8.3%) patients dropped out after V2, 38 (8.5%) patients quit after V3, and 37 patients (8.3%) discontinued treatment after V4. Moreover, patients were asked whether they took the drops daily during the treatment period, and 320 patients (74.1%) mentioned that they took the drops daily, while 112 did not.

### 3.4. Safety and Tolerability

**Figure 2:**
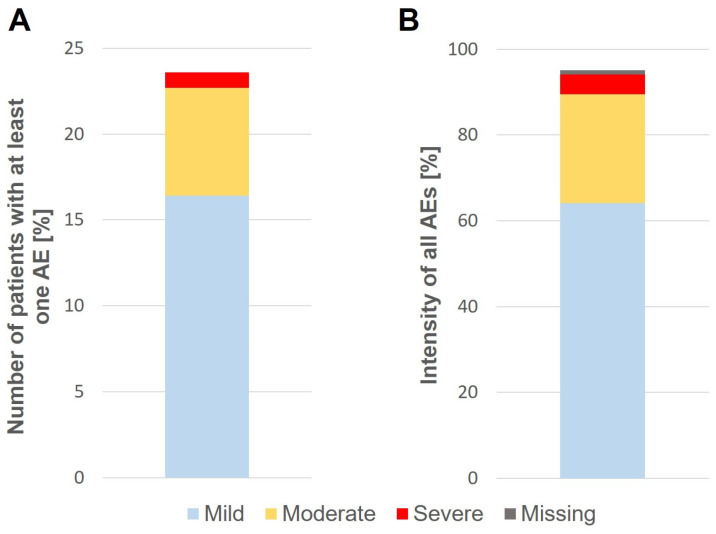
Overview of AE occurring during the 8-month treatment period. (**A**) Number of patients with at least one AE categorized by intensity (%). (**B**) Intensity of all reported AEs (%).

The data on reported AEs during the treatment period of 8 months are shown in Figure 2. AEs were graded by intensity as mild, moderate, or severe. Overall, 102 (23.6%) study participants treated with SBPE or STPE reported at least one AE during the treatment period. Most AEs were mild or moderate in severity. While 71 (16.4%) patients experienced at least one mild AE, 27 (6.3%) and 4 (0.9%) patients developed moderate or severe AEs, respectively. The number of all documented AEs (Figure 2B) was 351. The majority of these AEs were mild (225 equivalent to 64.1%). A total of 89 (25.4%) AEs were graded as moderate, and only 16 (4.6%) were considered severe. Twenty-one (6.0%) of the documented AEs were not graded by the reporter (Figure 2).

AEs were categorized into local or systemic reactions, and although the allergens of hazel, alder, and birch are highly homologous, the safety of SBPE and STPE was separately analyzed (Table 4). In both groups, the majority of AEs involved local reactions, while systemic reactions occurred less frequently. The AE intensity of local reactions was mainly mild (SBPE: 39 patients (18.8%) vs. STPE: 30 patients (13.4%)). Moderate local reactions occurred in 7 (3.4%) and 17 (7.6%) patients treated with SBPE or STPE, respectively. Severe local reactions were observed in only 1 (0.5%) SBPE-treated patient while 2 (0.9%) STPE-treated patients developed severe local reactions. The total number of all reported local reactions was comparable between the groups and accounted for 124 events in the SBPE group and 147 events in the STPE group. 

In SBPE-treated patients, 104 of these local reactions (83.9%) were graded as mild while 14 (11.3%) were moderate and only 3 (2.4%) were severe. Additionally, 3 local reactions (2.4%) in SBPE-treated patients were not graded by the reporter. In the STPE group, 98 local reactions (66.7%) were graded as mild. The number of moderate local reactions was 41 (27.9%), while 8 (5.4%) local reactions were reported as severe. 

Systemic reactions occurred in both groups less frequently compared with local reactions. While 9 patients (4.3%) in the SBPE group developed at least one systemic reaction, 18 (8.0%) STPE-treated patients suffered from at least one systemic reaction during this NIS. The AE grading revealed that grade 0 reactions occurred in 1 (0.5%) and grade 1 reactions in 2 (1%) SBPE-treated patients. More severe grade II and III reactions occurred in only 1 (0.5%) patient in each group. In the STPE subgroup, grade I and II reactions occurred more frequently as compared with the STPE-treated individuals. Nine patients (4%) suffered from grade I reactions, and five (2.2%) STPE-treated patients developed grade II reactions. Grade III reactions were not reported for the STPE group. Four (1.9%) patients in the SBPE group and three (1.3%) in the STPE group experienced systemic reactions, which were not graded by the reporter. In total, 7 patients reported cases of grade II and III systemic reactions. These reactions included 1 case of nausea, ear pruritus, rhinnorrhorea (grade III), 2 cases of breathing difficulties, circulatory problems, rhinnorrhorea (grade II), 3 cases of tightness in the chest area (grade II), 4 cases of asthma (grade II), 5 episodes of heart racing, itching (grade II), 6 cases of heartburn (grade II), 7 episodes of earache and breathing difficulties (grade II). In total, 57 systemic reactions were reported by the physicians during the 8-month treatment period. Twenty of these systemic reactions occurred in the SBPE group and 37 in the STPE group. Among patients treated with SBPE, 4 (20%) events were classified as grade 0 and I reactions, respectively. Two (10%) events were grade II, and three (15%) events were grade III reactions. Despite the fact that more systemic reactions occurred in STPE-treated patients, most of the AEs involved grade I reactions (20 events according to 54.1%). Grade 0 reactions were observed in 1 (2.7%) event, while 9 (24.3%) events were in grade II. Seven events (SBPE: 35.0%; STPE: 18.9%) were not graded by the reporter in each of the two groups. 

Additionally, 23 reactions of other kinds occurred in 13 patients (3%) during the NIS. These reactions could not be categorized as local or systemic reactions. In the SBPE group, only 2 patients suffered from other reactions, and in the STPE group, 11 patients developed at least one other reaction. However, only one reaction of the other kind was classified as severe, while the rest was classified as mild, moderate, or with missing information on the intensity (Table 4). 

Overall, no major differences in the safety of SBPE and STPE were observed, but patients treated with STPE tended to develop systemic reactions slightly more often than SBPE-treated patients.

Furthermore, we analyzed the occurrence of adverse events in the subgroup of patients characterized by asthma. We observed that the incidence of the development of adverse events was higher in the group of patients with asthma than in the group without asthma. At least one adverse event occurred in 24 (47.1%) patients with asthma, while only 78 (20.5%) patients without asthma had at least one adverse event. However, most of the adverse events represented mild or moderate adverse events.

### 3.5. Treatment Satisfaction

The treatment satisfaction of study participants is associated with the patient’s compliance [13,14]. Patients that completed all visits were asked about their treatment satisfaction with this SLIT. In this context, patients were only asked whether they were satisfied with the sublingual therapy, but we did not define any criteria for patient satisfaction. Therefore, the question about patient satisfaction focused more on the handling of the sublingual immunotherapy and patient compliance. 

As shown in Table 5, the majority were very satisfied or satisfied with the sublingual treatment (98.6%). Only 1.4% of the patients were unsatisfied or very unsatisfied with the treatment. However, treatment satisfaction data from patients who dropped out during this NIS were not available.

### 3.6. Change of Oral Allergy Syndrome (OAS)

**Figure 3:**
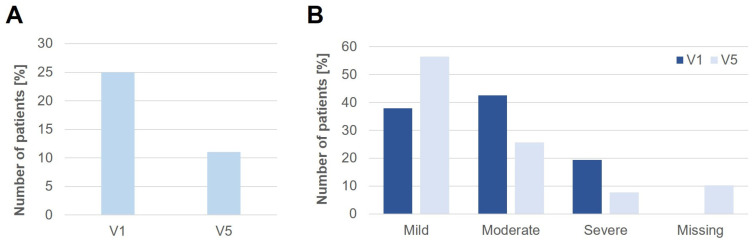
Influence of SLIT on oral allergy syndrome (OAS). (**A**) Percentage of patients suffering from OAS at V1 (before treatment) and at V5 (after 8 months of treatment). (**B**) OAS severity in affected patients at visits V1 and V5.

The number of patients with oral allergy syndrome (OAS) decreased after sublingual treatment with birch or tree pollen extracts. While 25% of the patients suffered from an OAS at V1, only 11.1% reported OAS symptoms after the treatment period of 8 months at V5 (Figure 3A). 

While the number of patients with mild OAS symptoms increased over time (V1 38.0% vs. V5 56.4%), the number of patients with moderate (V1 42.6% vs. V5 25.6%) and severe (V1 19.5% vs. V5 7.7%) OAS symptoms decreased (Figure 3B). No information about the severity was available at V5 for 10.3% of the patients. Altogether, these data suggest that sublingual treatment with SBPE/STPE might be able to reduce OAS symptoms in certain patients.

## 4. Discussion

Marketing authorization for sublingual birch (SBPE) and sublingual tree pollen (STPE) extracts was obtained from the PEI in 2018 after the efficacy was demonstrated in a clinical phase III study [7]. However, NIS maintains a vital role in supporting the safety of a new product post approval. This NIS was designed with the primary objective of collecting safety information during the daily clinical use of SBPE and STPE in Germany. 

The study population represented patients suffering from allergic rhinoconjunctivitis with or without asthma, who were treated with SLIT for their allergic symptoms. The number of female and male patients was nearly identical, and the number of patients treated with STPE and SBPE was also comparable. The inclusion and exclusion criteria were in accordance with the SPC [8] of the registered products.

During the 8-month treatment period with SBPE or STPE, a total of 102 patients (23.6%) of the study population experienced at least one AE. Most of these AEs were considered local reactions (69 patients (22.2%)), while systemic reactions were observed less frequently (27 patients (6.3%)). The AE intensity was rated as mild to moderate in most of the cases, and severe events were uncommon. Regarding all documented systemic reactions, the majority of cases were grade I reactions (24 events (42.1%). Grade II (11 events (19.3%)) and grade III reactions (3 events (5.3%)) occurred less frequently. The grade III reaction involved a case of nausea, ear pruritus, and rhinorrhea. According to the guidelines on acute therapy and management of anaphylaxis [15], this case might rather be classified as a grade II systemic reaction.

A subgroup analysis of patients with or without asthma revealed that patients with asthma had a higher incidence to develop at least one adverse event compared with patients without asthma. Although adverse events occurred more frequently in patients with asthma, these adverse events were mostly mild to moderate in intensity.

When compared with the data from the phase III clinical trial with SBPE [7], local and systemic reactions occurred less frequently than expected in this NIS. While 59.1% of the patients suffered from at least one local reaction in the clinical trial, only 22.2% of the patients in this NIS developed local reactions. Systemic reactions were also less frequently reported in the NIS (6.3%) compared with the clinical trial (17.8%). However, the severity of AEs was comparable between the two studies. A possible reason for the lower number of reported AEs in this NIS might be that the documentation in the eCRF was not monitored by a clinical research organization or a clinical monitor. Furthermore, no source data validation was performed as it is performed for clinical trials.

The treatment compliance in this NIS (71.0%) was lower when compared with the compliance in the clinical phase III study (99.0%) [7]. Both parameters—fewer AEs as well as lower compliance—suggest that noncompliant patients might have suffered from AEs that were not reported to the physicians, which resulted in the premature withdrawal of these patients from the study. Therefore, the actual number of AEs was probably higher than reported in this NIS. High-dose SLIT preparations often cause, especially at the start of the treatment, local or mild systemic reactions. These could influence treatment compliance in the daily life of the patients. In addition, the reason that patients do not usually have any contact with the physician for many weeks during SLIT could result in lower treatment compliance in the NIS compared with clinical trials. 

Another important compliance factor is the satisfaction of patients with the treatment, because unpleasant experiences, such as strong side effects or complicated handling, can impact the patient’s behavior during therapy [13,14]. In this NIS, 134 (98%) patients who completed all visits were very satisfied or satisfied with the sublingual treatment, while only 5 patients were not satisfied. Unfortunately, the satisfaction of patients who dropped out before the end of the NIS was not available. These patients might not have been satisfied with the sublingual treatment. Data on patient adherence at a large single German allergy center showed that most patients who discontinue AIT do so during the first year of therapy [16]. In a systematic review, Makatsori et al. analyzed 81 studies and observed for SLIT trials a dropout rate of 14% [17]. In contrast, Kiel et al. showed, using real-life compliance data, that only 7% of SLIT users reached the minimally required treatment duration of 3 years [18].

In particular, treatment compliance during SLIT compared with SCIT was widely debated in previous research. While Inconvaria et al. found similar compliance rates for both therapies, ranging from 75% to 90% [19], other studies demonstrated higher treatment compliance for SCIT compared with SLIT using real-world compliance data [18,20]. Of note, most data on compliance are from clinical trials and are not appropriate for predicting treatment adherence in a real-world setting [21]. Relevant studies regarding treatment compliance are summarized in Section 6.4 of the S2k guideline on AIT from 2022. In addition, the S2k guideline on AIT lists different measures, such as management in practice and clinic, patients-specific requirements as well as patient information and instruction, to improve treatment adherence [22]. In order to ensure adequate therapeutic adherence and compliance, improved recall systems are necessary for SLIT as well as for SCIT [23]. We also believe that a contact person for AIT patients could be valuable as another measure in practice and clinic to answer any questions or uncertainties patients may have. However, patient information and instruction measures are, in our opinion, at least as important as measures in practice and clinic. It is important to educate patients about the possibility of the occurrence of AEs in the first weeks of SLIT. In addition to appropriate patient education, a better understanding of primary and secondary preventative AIT effects by the patient might also increase the number of compliant patients in SLIT. Finally, patient-specific requirements to improve therapy adherence, such as perception of the disease burden and compatibility of AIT with the patient’s daily life, may also have a major impact on treatment adherence [22]. 

An advantage of liquid birch and tree pollen allergen extracts is that slower individual up-dosing is possible in case of AE occurrence. Instead of adding one drop each consecutive day until the maintenance dose of five drops per day is reached, it is possible to increase the number of drops over a longer time period to ensure a gentle acclimatization of the immune system to the allergen. Another advantage is that less than 5 drops can be defined as an individual maintenance dose. During the pollen season, it is possible to temporarily reduce the maintenance dose in case of severe allergic symptoms. 

Regarding compliance, AIT with subcutaneous products may be superior to the sublingual treatment since the subcutaneous injection requires regular visits to the physician’s office, which ensures regular and frequent patient-physician interaction. 

The influence of AIT on OAS has been investigated by different research groups, and the potential treatment effect of SLIT on pollen food syndrome caused by fruits (apples) was confirmed [24,25,26,27]. Approximately 47–70% of all patients allergic to birch pollen develop an OAS during their lifetime [28]. OAS is characterized by the onset of oropharyngeal symptoms following the consumption of certain foods, such as apples. OAS is caused by cross-reactive IgE antibodies that recognize the major birch pollen allergen Bet v 1 as well as food proteins. Since we observed a reduction in OAS symptoms after treatment with SBPE and STPE, our results suggest that patients with moderate or severe OAS, in particular, might benefit from the sublingual therapy. However, some questions, e.g., the long-lasting improvement of OAS after the termination of SLIT, need further investigation. 

## 5. Conclusions

The NIS described here showed good safety and tolerability of SBPE and STPE. No major differences in safety and tolerability were observed in patients treated with these products. The incidence of AEs was lower in this NIS as compared with the data of the clinical phase III study, but the intensity of reported AEs was comparable. Systemic reactions were uncommon. No new information with regard to the safety of SBPE and STPE pollen extract became apparent in this NIS.

## Figures and Tables

**Table 1:** Overview of visits carried out during the NIS.

Visits	Purpose	Description
V1	Screening	Review of inclusion and exclusion criteria and collection of demographic data, medical history, concomitant diseases, and medication use
V2	Administration	Initial administration of sublingual treatment
V3	Maintenance	Documentation of treatment adherence and the occurrence AEs
V4		
V5	Final visit	Recording of AEs and patient satisfaction

**Table 2:** Summary of patient characteristics at the first visit (V1) (*n* = 432).

Characteristics	*n* (%)
*Gender*	
Male	189 (43.8)
Female	243 (56.3)
*Age*	
Mean (SD) [years]	44.3 (14.6)
Median (Min–Max)	44.0 (19.0–83.0)
*Asthma*	
Yes	51 (11.8)
No	381 (88.2)
*Oral allergy syndrome*	
Yes	108 (25.0)
No	324 (75.0)
*Treatment*	
SBPE	208 (48.1)
STPE	224 (51.9)

**Table 3:** The number of patients who reached the maintenance dose of 5 drops during the 8-month treatment period with SBPE or STPE and the number of patients who reached 5 drops within 5 days.

	Yes *n* (%)	No *n* (%)	Total *n* (%)
Maintenance dose	385 (96.0)	16 (4.0)	401 (100.0)
Maintenance dose within 5 days	363 (94.3)	22 (5.7)	385 (100.0)

**Table 4:** Summary of local reactions (LR), systemic reactions (SR), and other reactions (OR) in all patients treated with either SBPE or STPE.

Parameters		SBPE (*n* = 208) *n* (%)	STPE (*n* = 224) *n* (%)	Total (*n* = 432) *n* (%)
Patients with at least one LR	Σ	47 (22.6)	49 (21.9)	96 (22.2)
Strongest intensity	Mild	39 (18.8)	30 (13.4)	69 (16.0)
	Moderate	7 (3.4)	17 (7.6)	24 (5.6)
	Severe	1 (0.5)	2 (0.9)	3 (0.7)
Number of all LR	Σ	124 (100)	147 (100)	271 (100)
Intensity	Missing	3 (2.4)	0 (0)	3 (1.1)
	Mild	104 (83.9)	98 (66.7)	202 (74.5)
	Moderate	14 (11.3)	41 (27.9)	55 (20.3)
	Severe	3 (2.4)	8 (5.4)	11 (4.1)
Patients with at least one SR	Σ	9 (4.3)	18 (8.0)	27 (6.3)
Strongest intensity	Missing	4 (1.9)	3 (1.3)	7 (1.6)
	Grade 0	1 (0.5)	1 (0.4)	2 (0.5)
	Grade I	2 (1.0)	9 (4.0)	11 (2.5)
	Grade II	1 (0.5)	5 (2.2)	6 (1.4)
	Grade III	1 (0.5)	0 (0.0)	1 (0.2)
Number of all SR	Σ	20 (100.0)	37 (100.0)	57 (100.0)
Intensity	Missing	7 (35.0)	7 (18.9)	14 (24.6)
	Grade 0	4 (20.0)	1 (2.7)	5 (8.8)
	Grade I	4 (20.0)	20 (54.1)	24 (42.1)
	Grade II	2 (10.0)	9 (24.3)	11 (19.3)
	Grade III	3 (15.0)	0 (0.0)	3 (5.3)
Patients with at least one OR	Σ	2 (1.0)	11 (4.9)	13 (3.0)
Strongest intensity	Missing	0 (0.0)	3 (1.3)	3 (0.7)
	Mild	1 (0.5)	3 (1.3)	4 (0.9)
	Moderate	1 (0.5)	4 (1.8)	5 (1.2)
	Severe	0 (0.0)	1 (0.4)	1 (0.2)
Number of all OR	Σ	2 (100.0)	21 (100.0)	23 (100.0)
Intensity	Missing	0 (0.0)	10 (47.6)	10 (43.5)
	Mild	1 (50.0)	4 (19.0)	5 (21.7)
	Moderate	1 (50.0)	6 (28.6)	7 (30.4)
	Severe	0 (0.0)	1 (4.8)	1 (4.4)

**Table 5:** Summary of treatment satisfaction at the last visit (V5) in SBPE and STPE-treated patients.

Treatment Satisfaction	*n* (%)
Very satisfied	208 (58.9)
Satisfied	140 (39.7)
Unsatisfied	4 (1.1)
Very unsatisfied	1 (0.3)
Total	353 (100)

## Data Availability

Data is unavailable due to privacy or ethical restrictions.
